# *CASP8* SNP D302H (rs1045485) Is Associated with Worse Survival in *MYCN*-Amplified Neuroblastoma Patients

**DOI:** 10.1371/journal.pone.0114696

**Published:** 2014-12-11

**Authors:** Ali Rihani, Bram De Wilde, Fjoralba Zeka, Geneviève Laureys, Nadine Francotte, Gian Paolo Tonini, Simona Coco, Rogier Versteeg, Rosa Noguera, Johannes H. Schulte, Angelika Eggert, Raymond L. Stallings, Frank Speleman, Jo Vandesompele, Tom Van Maerken

**Affiliations:** 1 Center for Medical Genetics, Ghent University, Ghent, Belgium; 2 Department of Pediatric Hematology, Oncology and Stem Cell Transplantation, Ghent University Hospital, Ghent, Belgium; 3 Département de pédiatrie, hémato-oncologie, SUHOPL- CHC (Service Universitaire d′HématoOncologie Pédiatrique Centre Hospitalier Chrétien) Espérance, St Nicolas Belgium; 4 Neuroblastoma Laboratory, Onco/Hematology Laboratory, Department of Women's and Children's Health, University of Padua, Pediatric Research Institute, Fondazione Città della Speranza, Padua, Italy; 5 Lung Cancer Unit, IRCCS (Istituto di Ricovero e Cura a Carattere Scientifico), Azienda Ospedaliera Universitaria San Martino – IST (Istituto Nazionale per la Ricerca sul Cancro), Genoa, Italy; 6 Departement of Human Genetics, Academic Medical Center, University of Amsterdam, Amsterdam, the Netherlands; 7 Department of Pathology, Medical School, University of Valencia, Valencia, Spain; 8 German Cancer Consortium (DKTK), Germany; 9 Translational Neuro-Oncology, West German Cancer Center, University Hospital Essen, University Duisburg-Essen, Essen, Germany; 10 German Cancer Research Center (DKFZ), Heidelberg, Germany; 11 Department of Pediatric Oncology and Haematology, University Children's Hospital Essen, Essen, Germany; 12 Centre for Medical Biotechnology, University Duisburg-Essen, Essen, Germany; 13 Department of Molecular and Cellular Therapeutics, Royal College of Surgeons in Ireland, and National Children′s Research Centre, Dublin, Ireland; ENEA, Italy

## Abstract

**Background:**

Neuroblastoma is a pediatric cancer that exhibits a wide clinical spectrum ranging from spontaneous regression in low-risk patients to fatal disease in high-risk patients. The identification of single nucleotide polymorphisms (SNPs) may help explain the heterogeneity of neuroblastoma and assist in identifying patients at higher risk for poor survival. SNPs in the TP53 pathway are of special importance, as several studies have reported associations between TP53 pathway SNPs and cancer. Of note, less than 2% of neuroblastoma tumors have a *TP53* mutation at diagnosis.

**Patients and Methods:**

We selected 21 of the most frequently studied SNPs in the TP53 pathway and evaluated their association with outcome in 500 neuroblastoma patients using TaqMan allelic discrimination assays.

**Results and Conclusion:**

We investigated the impact of 21 SNPs on overall survival, event-free survival, age at diagnosis, *MYCN* status, and stage of the disease in 500 neuroblastoma patients. A missense SNP in exon 10 of the *CASP8* gene SNP D302H was associated with worse overall and event-free survival in patients with *MYCN*-amplified neuroblastoma tumors.

## Background

Neuroblastoma (NB) is a complex and heterogeneous pediatric cancer that can manifest with tumors that regress spontaneously, and also with tumors that metastasize and acquire resistance to therapy, leading to severe illness and death [1]. Currently, the treatment protocols depend on the risk stratification at diagnosis, which in turn is based on the clinico-genetic features of the patient and the tumor. The risk stratification system has been improved over the past decades [2]; however, it remains imperative to further exploit the underlying biology of clinico-genetic features of the disease for the sake of tailoring therapies to defined patient populations. Inter-individual variability in response to therapy can result from single nucleotide polymorphisms (SNPs) in critical genes involved in cell cycle control and induction of apoptosis [3]. Some SNPs have proven their usefulness in serving as prognostic markers in development of targeted therapies, or defining patient populations in terms of potential response to conventional therapies. One of the most critical genes that acts as a guardian of the genome and also mediates response to therapy is *TP53* [4]. *TP53* is mutated in more than 50% of human cancers [5]; however, less than 2% of NB tumors harbor a *TP53* mutation at diagnosis [6]. This suggests that other players in the TP53 pathway such as SNPs may play a role in NB.

Genetic polymorphisms in TP53 pathway components have been targeted for personalizing current therapies and developing new treatment modalities [7]. However, only a limited number of studies have focused on the association between SNPs in TP53 pathway genes and NB. Therefore, we selected 21 SNPs in fifteen TP53 pathway associated genes (Table 1), which have been reported to be involved in susceptibility to several cancer types. SNP selection was primarily based on a study that identified potential SNPs involved in TP53 stress response using publicly available genotypes and drug response data from the NCI60 human tumor cell lines [8]. NCI60 is a well-characterized panel of 60 human cancer cell lines from different cancer entities with publicly available anti-cancer drug screening data [9] and genotype data from 100,000 SNPs in cancer related genes [10], [11]. We expanded this selection to include SNPs in TP53 pathway associated genes that have been reported elsewhere to be involved in cancer susceptibility [4], [12]–[15]. The 21 SNPs are found in genes involved in DNA damage response, apoptotic response, cell cycle regulation, direct regulation of TP53, or the *TP53* gene itself (Table 1). We genotyped these SNPs in 500 NB cases and investigated the association between these SNPs and overall and event-free survival, age at diagnosis, *MYCN* status, and the stage of the disease of NB patients.

**Table 1:** 21 SNPs in 15 p53 pathway genes.

Gene symbol	Reference SNP ID	Description	Residue	Ref	Taqman assay ID	SNP frequency
*ATM*	rs1800054	missense C>G	Ser49Cys	[4]	C___2283268_20	0.018
*CASP8*	rs1045485	missense G>C	Asp302His	[4]	C___8823877_20	0.101
*CDKN1A*	rs1801270	missense C>A	Ser31Arg	[4]	C_____14977_20	0.092
*TP53*	rs1042522	missense C>G	Pro72Arg	[4]	C___2403545_10	0.291
*CCNG1*	rs2069347	Intronic T>C		[8]	C___2000410_20	0.407
*CD44*	rs187115	Intronic G>A		[8]	C____779820_10	0.368
*YWHAQ*	rs6734469	Intronic A>G		[8]	C__29724290_10	0.456
*PIAS1*	rs1027154	Intronic G>C		[8]	C___1935268_20	0.085
*PPP2R2B*	rs319217	Intronic A>G		[8]	C___3065531_30	0.403
*PPP2R2B*	rs319227	Intronic T>G		[8]	C____803346_10	0.361
*CSE1L*	rs2426127	Intronic C>T		[8]	C__16230087_10	0.288
*KDR*	rs2168945	Intronic T>G		[8]	C___1673863_10	0.350
*MDM2* (285)	rs117039649	Intronic G>C		[12]		0.093
*MDM2* (309)	rs2279744	Intronic T>G		[4]		0.376
*MDM2* (344)	rs1196333	Intronic T>A		[13]		0.05
*MDM2* (354)	rs769412	Synonymous A>G		[14]		0.076
*MDM4*	rs4245739	3′UTR A>C		[15]	C__11623776_10	0.264
*TP53*	rs78378222	3′UTR A>C		[36]	AHKASE2	0.050
*CDKN1B*	rs34330	5′ UTR T>C		[4]	C___2402292_10	0.244
*TP73*	rs2273953	5′ UTR C>T		[4]	C__16180357_10	0.197
*TP73*	rs1801173	5′ UTR C>T		[4]	C__16180356_10	0.169

## Materials and Methods

### Patients

The study group comprised 500 NB patients who were evaluated according to the International NB Staging System (INSS) [16]. All patients included in this study provided written informed consent for anonymous use of the samples for research related to neuroblastoma biology. Approval for this study was granted by the medical ethical committee of Ghent University Hospital. Registration number: B670201111331.

### Statistical methods

Comparisons of Kaplan-Meier survival curves between different genotype groups were done using the log-rank test. Survival time was defined as the time from diagnosis until death from the disease or until the date of the last follow-up of living patients. Patient samples were divided into two groups based on SNP status (wild-type versus homozygous or heterozygous for the minor allele). Comparisons of age at diagnosis between the different groups were done using the Mann-Whitney *U* test. Association of the SNPs with *MYCN* status or stage of the disease was done using Pearson's Chi-square test. Statistical tests were two-sided and results with corrected *p*-values (*q*-values) <0.05 were considered statistically significant. All statistical analyses were conducted using SPSS version 20 software. All statistical analyses were followed by Benjamini-Hochberg multiple testing correction in R version 3.0.2 using “stats” statistical package.

### SNP selection and genotyping

SNPs in potentially important TP53 pathway genes were selected from previously reported studies [4], [8], [12]–[15]. The protein products of these genes function at different levels of the TP53 pathway. *MDM2* SNPs (285, 309, 344, and 354) were genotyped by Sanger sequencing and the remaining 17 SNPs in TP53 pathway genes were genotyped using the TaqMan allelic discrimination assay. NB tumor DNA was first amplified using illustra GenomiPhi V2 DNA amplification kit (GE Healthcare Life Sciences, Buckinghamshire UK) according to the manufacturer's protocol. Genotyping was carried out using 10 ng of amplified genomic DNA per reaction, with 2.5 µL of 2X TaqMan Genotyping Master Mix and 160 nM primers (Life Technologies, Merelbeke, Belgium) in a 5 µL reaction volume. All genotypes were determined by endpoint calling on an ABI 7900 real-time PCR instrument (Life Technologies, Merelbeke, Belgium). PCR cycling conditions were 50°C for 2 min, 95°C for 10 min, followed by 40 cycles of 92°C for 15 s and 60°C for 1 min. Sanger sequencing was carried out by PCR amplification and subsequent sequencing. The primers used for PCR amplification were tagged with universal M13 sequencing tags (underlined): forward primer CACGACGTTGTAAAACGAC TGGCTTTGCGGAGGTT and reverse primer CAGGAAACAGCTATGACC TCGGAACGTGTCTGAACTT. The PCR was performed in a 25 µl reaction mix using 20 ng of DNA, 1x KAPA2G buffer, 2.5 mM MgCl_2_, 0.2 mM dNTP mix, 0.5 µM forward and reverse primers, and 1 U KAPA2G Robust HotStart DNA polymerase. PCR conditions were as follows: initial denaturation at 94°C for 4 min, denaturation at 94°C for 20 s, primer annealing at 68°C for 15 s, and extension at 72°C for 1 min for 12 cycles. This was followed by another 34 cycles: denaturation at 94°C for 30 s, primer annealing at 55°C for 30 s, and extension at 72°C for 30 s. Finally, an extension step at 72°C for 4 min was performed, followed by cooling at 15°C for 1 min. The size of the amplified PCR product was verified using Caliper Labchip GX (PerkinElmer, Waltham, MA, USA). The sequencing was done using Sanger DNA sequencing service (Genewiz, South Plainfield, NJ, USA). The generated data was analyzed using Seqscape software v.2.7 (Life Technologies, Ghent, Belgium). 18 SNPs were genotyped in 31 NB cell lines (amplified and non-amplified DNA) to ensure the quality of the DNA amplification procedure.

### Reverse transcription quantitative polymerase chain reaction (RT-qPCR)

RT-qPCR was performed on duplicate samples using 5 ng of template cDNA, 2.5 µL of 2x Soo Advanced Reaction Mix (Roche), and 0.25 µL of a 5 µM solution of each primer in a 5 µL total reaction volume. The PCR conditions were as follows: 10 min at 95°C, followed by 45 cycles of denaturation (10 s at 95°C), and elongation (45 s at 60°C) using LightCycler480 (Roche). Primers for caspase-8 and the reference genes (Alu-Sq, HMBS, HPRT1, SDHA, and UBC) were designed using an in-house web tool (www.primerXL.org). The sequence of the primers can be found in Table S1 in S1 File. Relative expression of caspase 8 was quantified using Biogazelle's qbase+ qPCR data-analysis software version 2.y [17]

## Results

### *CASP8* SNP D302H and survival in NB patients

**Figure 1:**
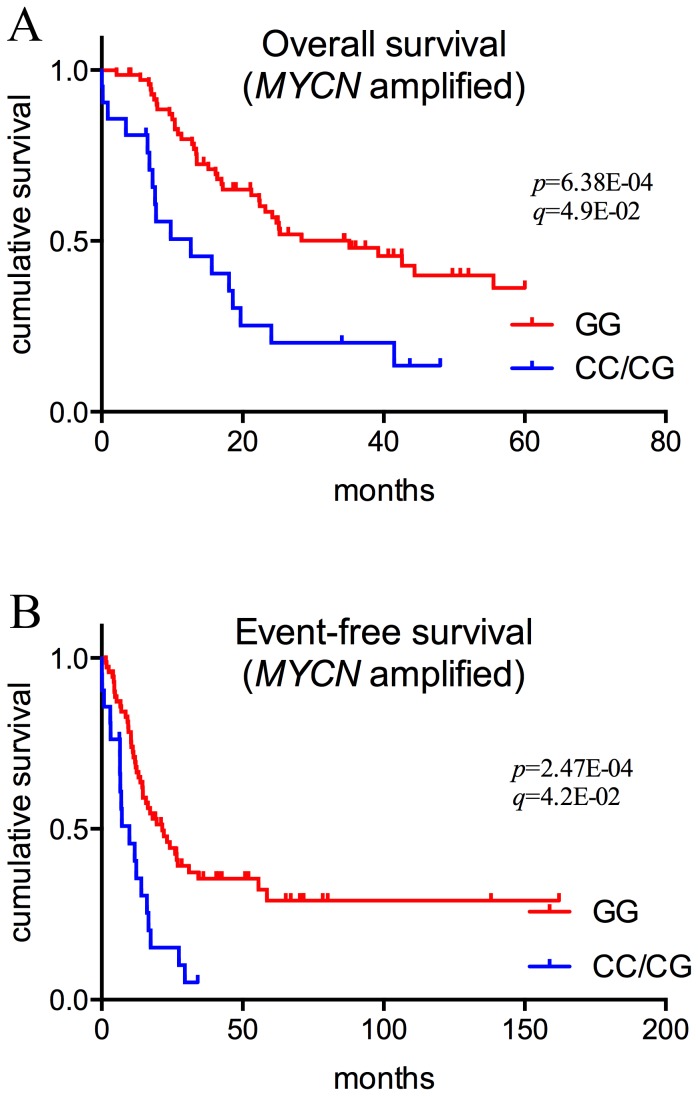
Overall and event-free survival of NB patients by *CASP8* SNP D302H. Comparison of Kaplan-Meier survival curves between different genotypes of *CASP8* SNP D302H. Overall survival of *MYCN*-amplified NB patients (**A**), event-free survival of *MYCN*-amplified NB patients (**B**). Raw *P*-values were calculated by the log rank test. *q*-values are adjusted *p*-values after Benjamini-Hochberg multiple testing correction.

We used whole genome amplified DNA from tumors of 500 NB patients to genotype 21 SNPs in fifteen TP53 pathway genes (Table 1) using hydrolysis probe based qPCR genotyping assays for 17 SNPs, and Sanger sequencing for four *MDM2* SNPs (285, 309, 344, and 354). The clinical features of the patients are shown in Table 2. The study population consisted mostly of European patients from Caucasian origin. We first validated the approach by genotyping the SNPs on whole genome amplified and non-amplified DNA from 31 NB cell lines, and results showed that all SNPs were genotyped correctly (Table S2 in S1 File). In patients, *CASP8* SNP D302H was the only SNP that showed an association with worse overall (OS) (*p* = 0.0006; multiple testing corrected *p*-value, *q*-value = 0.049) and event-free survival (EFS) (*p* = 0.0002; multiple testing corrected *p*-value, *q*-value = 0.042) in NB patients with *MYCN* amplification (Fig. 1, Table 3). The stratified survival analyses of all the other SNPs are shown in table S3 in S1 File. To confirm that there is no difference in the SNP status between the tumor and the germline of the patients, we collected 41 representative blood samples from the patients in our cohort and genotyped them using the same TaqMan assay for the *CASP8* SNP D302H. Our results show that all the 41 patients have exactly the same SNP status in their blood as in their tumor (data not shown). This additional analysis makes it highly unlikely that different SNP status exists in the tumor compared to the blood of the patients. We performed also multivariate Cox proportional hazard analysis and investigated the effect of *CASP*8 SNP D302H on the survival of neuroblastoma patients taking into account the different clinical covariates (stage of the disease, *MYCN* status, and the age at diagnosis). Our results show that *CASP*8 SNP D302H is an independent prognostic factor (Table S4 and S5 in S1 File)

**Table 2:** Clinical characteristics of the patients.

		Number	Percentage
Age at diagnosis			
	>12 months	296	59.2
	<12 months	204	40.8
Stage			
	1	103	20.6
	2	77	15.4
	3	92	18.4
	4	183	36.6
	4s	45	9
* MYCN*			
	Not amplified	406	81.2
	Amplified	94	18.8
Survival			
	Alive	380	76
	Dead	120	24
Group			
	Ghent	138	27.6
	Amsterdam	74	14.8
	Essen	35	6.85
	Valencia	71	14.2
	Dublin	108	21.6
	Genova	74	14.8
	Total	500	100

**Table 3:** Survival of NB patients by *CASP8* D302H status.

Overall survival	GG		CC/CG				
*CASP* D302H	Dead	Censored	Dead	Censored	Chi-Square	*P-*values	*q*-values
All stages	88	321	32	59	6.57	0.0079	**0.277**
Stage 4	75	70	25	13	1.26	0.127	**0.901**
Stages (1,2,3,4s)	13	251	7	46	4.41	0.035	**0.594**
MNA	38	35	17	4	11.7	0.0006	**0.049**
MNN	50	286	15	55	1.11	0.251	**0.901**

Event-free survival	GG		CC/CG				
*CASP8* D302H	Event	Censored	Event	Censored	Chi-Square	*P-*values	*q*-values
All stages	122	287	39	52	3.81	0.0456	**0.638**
Stage 4	88	57	29	9	1.4	0.136	**0.901**
Stages (1,2,3,4s)	34	230	10	43	0.834	0.355	**0.901**
MNA	44	29	19	2	13.4	0.0002	**0.042**
MNN	78	258	20	50	0.157	0.665	**0.919**

Raw *P*-value is calculated by Log Rank (Mantel-Cox) test.

*q*-value is the adjusted *p*-value after Benjamini-Hochberg multiple testing correction.

Censored: The patient was alive or did not have an event until the last date of follow-up.

MNA: *MYCN*-amplified.

MNN: *MYCN* not amplified.

### Impact of SNPs on age at diagnosis and association with the stage of the disease and *MYCN* status

We investigated the correlation between 21 SNPs and NB onset in a non-stratified analysis (all subgroups) and also in an analysis stratified by *MYCN* status or by clinical stage (localized vs. stage 4). *CASP8* SNP D302H was not correlated to age at diagnosis (Table S6 in S1 File). The same results were obtained for the remaining SNPs (data not shown). We also investigated whether *CASP8* SNP D302H or any of the 21 SNPs is associated with the *MYCN* status or stage of the disease. *CASP8* SNP D302H was not associated with *MYCN* status or stage of the disease (Table S7 in S1 File). Similar results were obtained for the remaining SNPs (data not shown).

### *CASP8* SNP D302H and expression of caspase 8

**Figure 2:**
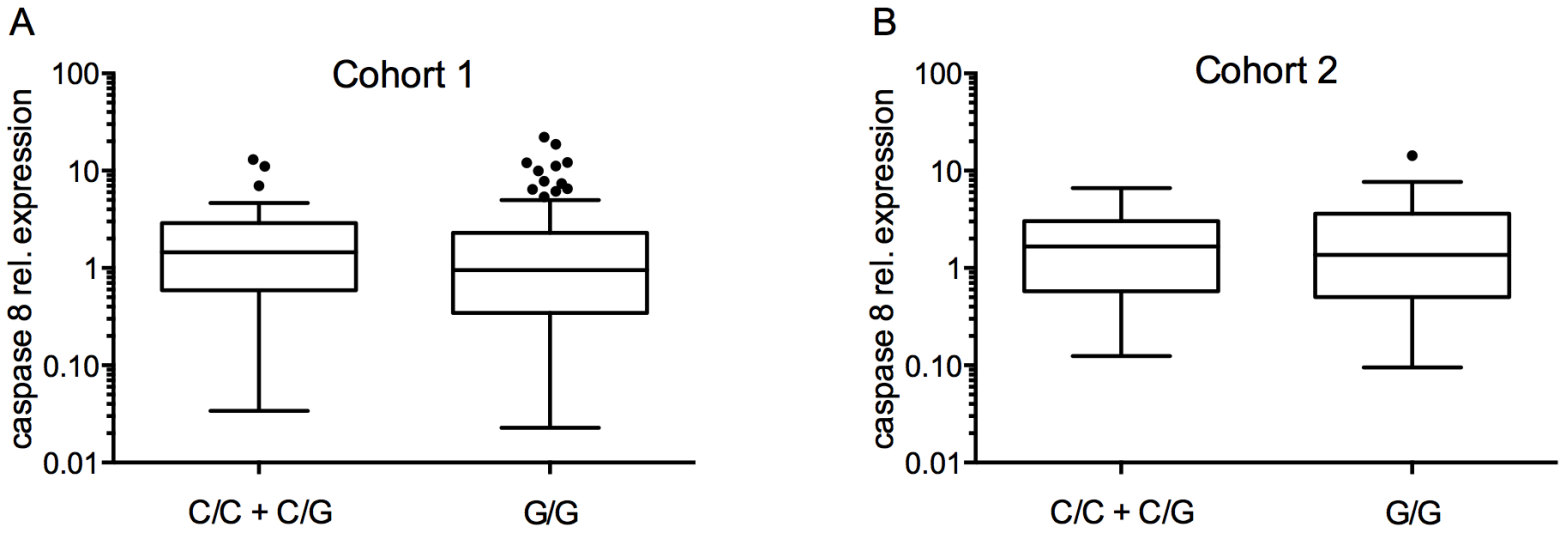
Expression of caspase-8 by *CASP8* SNP D302H. Comparison of caspase-8 expression between the different genotype groups of *CASP8* SNP D302H. Cohort 1 (**A**), Cohort 2 (**B**). *P*-values were calculated by the Mann-Whitney *U* test. *q*-values are adjusted *p*-values after Benjamini-Hochberg multiple testing correction.

Since decreased expression of caspase 8 is a recurrent event in NB [18], we tested whether *CASP8* SNP D302H could affect the expression of caspase 8. We measured caspase 8 mRNA expression in two independent cohorts of NB tumor samples. Cohort 1 included 162 samples treated according to the International Society of Pediatric Oncology Europe Neuroblastoma Group (SIOPEN, https://www.siopen-r-net.org/). Cohort 2 included 161 samples from the Neuroblastoma Research Consortium (NRC), which is a collaboration between several European NB research groups. All samples from cohorts 1 and 2 were derived from patients that had been included in the entire cohort of 500 patients analyzed in Table 1. We found that *CASP8* SNP D302H had no effect on caspase 8 mRNA expression levels, regardless of *MYCN* status or the stage of the disease (Table 4, Fig. 2).

**Table 4:** Distribution of caspase-8 expression by *CASP8* D302H.

Cohort 1	GG		CC/CG				
*CASP8* D302H	N	Mean Rank	N	Mean Rank	*U*	*P*-values	*q*-values
All stages	132	79.2	30	91.8	1670	0.184	0.897
Stage 4	30	18.9	7	19.5	102	0.894	0.974
Stages (1,2,3,4s)	102	60.8	23	72.7	951	0.156	0.897
MNA	23	13.4	4	17.5	32	0.372	0.897
MNN	109	26	26	25	1270	0.422	0.897

Cohort 2	GG		CC/CG				
*CASP8* D302H	N	Mean Rank	N	Mean Rank	*U*	*P*-values	*q*-values
All stages	127	80.6	33	82.7	2100	0.817	0.948
Stage 4	51	31.9	14	390	300	0.207	0.897
Stages (1,2,3,4s)	76	49.4	19	42.5	618	0.333	0.897
MNA	21	12.9	9	21.6	39.5	0.372	0.897
MNN	106	66.8	24	62.8	1250	0.639	0.916

Raw *P*-value is calculated by Mann-Whitney *U* test.

*q*-value is the adjusted *p*-value after Benjamini-Hochberg multiple testing correction.

## Discussion

NB is a heterogeneous disease with a broad clinical spectrum ranging from spontaneous regression and excellent outcome in low-risk patients to widely disseminated disease and poor survival in high-risk patients. The phenomenon of spontaneous NB regression has been attributed to successful delayed apoptosis of tumor cells, whereas progression towards uncontrolled proliferation and tumor formation largely reflects defects in the apoptotic process [19]. Evasion of growth suppressors such as TP53 is a hallmark of tumor formation and development [20], and functional inactivation of the TP53 pathway could be achieved either via mutations in the *TP53* gene itself or anywhere along the TP53 regulatory network. Because *TP53* is rarely mutated in NB at the time of diagnosis (<2%) [21], other components of the TP53 pathway could be responsible for circumvention of this fundamental antitumor barrier. Numerous reports have suggested that germline alterations in *TP53* and TP53 pathway genes are associated with cancer risk and clinical outcomes [4]. Several SNPs that predispose to NB have been identified by genome-wide association studies (GWAS). The SNPs most highly associated with increased NB susceptibility and poor outcome were found in *HACE1* (rs4336470) [22], *LIN28* (rs17065417) [22], [23], *BARD1* (rs6435862, rs3768716) [24], *LMO1* (rs110419) [25], *DUSP12* (rs1027702), *DDX1* (rs2619046), *IL31RA* (rs10055201), and *HSD17B12* (rs11037575) [26]. However, despite the power of GWAS for identifying new predisposing SNPs, these studies still lack the ability to identify all SNPs and there is a high probability of false negatives [27]. Rather than choosing a GWAS approach with complex data analysis, we decided to conduct a focused study to examine particular SNPs in the TP53 pathway that are thought to influence susceptibility to cancer [4], [8], [12]–[15]. Our study represents the largest targeted SNPs study in a large cohort of 500 NB patients. We investigated the association of 21 SNPs in fifteen TP53 pathway associated genes with the survival, age at diagnosis, stage of the disease, and *MYCN* status of 500 NB patients. We did multiple testing correction using Benjamini-Hochberg method and considered only the *q*-values (corrected *p*-values) less than 0.05 as significant. Our results showed that one SNP, *CASP8* SNP D302H (rs1045485) was associated with worse overall and event-free survival in NB patients with *MYCN* amplification. Of note, we analyzed 41 representative blood samples from the patients in our cohort and observed that all these patients have the same SNP status in their blood as in their tumor (data not shown), indicating that *CASP8* SNP D302H is a germline risk factor for NB patients with *MYCN* amplification. *CASP8* SNP D302H is a missense variant located in exon 10 of the *CASP8* gene. *CASP8* encodes for inactive procaspase 8 that is later activated by dimerization to form caspase 8. Caspase 8 plays an important role in execution of the extrinsic cell death pathway (death receptor-mediated killing) and is involved in several other cell signaling pathways [28]. Down-regulation of caspase 8 is a well-characterized apoptotic defect in NB tumors [18]. *CASP8* is located on 2q33, a region frequently associated with loss of heterozygosity (LOH) in NB [29]. Down-regulation of caspase 8 can be achieved via deletion or epigenetic silencing, preferentially in *MYCN*-amplified NB tumors [30]. A recent study has shown that loss of caspase 8 expression in a Th-MYCN/caspase-8 deleted mouse model significantly enhanced development of advanced NB and metastasis to the bone marrow [31]. Although these two events (*MYCN* amplification and reduced caspase 8 expression) are closely related in NB, it has been shown that MYCN overexpression does not affect the methylation status or expression of *CASP8* [32]. We investigated the effect of *CASP8* SNP D302H on caspase 8 mRNA expression in two independent cohorts. Our results showed that *CASP8* SNP D302H had no effect on caspase 8 expression; being a non-synonymous SNP, it may therefore have a possible role in the activation of procaspase 8 or in the interaction of caspase 8 with elements that form the death-inducing signaling complex (DISC). *CASP8* SNP D302H encodes an amino acid residue that is localized on the external surface of procaspase 8 and that could affect the processing of procaspase 8 or caspase-8 interaction with other apoptosis regulators. However, until this is confirmed, the effect of this SNP on caspase 8 activity remains elusive. The well-known *MDM2* SNP309 was also included in our targeted analysis of 21 SNPs. Previous studies reported an association of this SNP with neuroblastoma [33], [34], and other cancers [13]; however, we have recently shown that this SNP is not associated with neuroblastoma. Ethnicity of the studied populations, statistical power, or genotyping data quality could be a reason for this discrepancy [35].

## Conclusions

In conclusion our study shows that SNP D302H, a missense SNP in exon 10 of *CASP8* gene, is associated with worse overall survival and event-free survival in NB patients with *MYCN* amplification.

## Supporting Information

S1 File **Supporting tables.** S1 Table, RT-qPCR primers. S2 Table, Comparison of genotyping data on amplified DNA (A) and non-amplified DNA (NA) for 18 SNPs in 31 NB cell lines. S3 Table, Stratified survival analysis of all the SNP. S4 Table, multivariate cox proportional hazards analysis (overall survival data). S5 Table, multivariate proportional hazards analysis (event-free survival data). S6 Table, *CASP8* SNPD302H and age at diagnosis. S7 Table, *CASP8* SNPD302H association with Stage 4 and *MYCN* status.
(DOCX)
