# Supplementary material for: CASP8 SNP D302H (rs1045485) Is Associated with Worse Survival in MYCN-Amplified Neuroblastoma Patients
Source: PLoS One. 2014 Dec 11;9(12):e114696. doi: 10.1371/journal.pone.0114696 (PMC4263607; doi:10.1371/journal.pone.0114696)
Supplement: S1 File — Supporting tables. S1 Table, RT-qPCR primers. S2 Table, Comparison of genotyping data on amplified DNA (A) and non-amplified DNA (NA) for 18 SNPs in 31 NB cell lines. S3 Table, Stratified survival analysis of all the SNP. S4 Table, multivariate cox proportional hazards analysis (overall survival data). S5 Table, multivariate proportional hazards analysis (event-free survival data). S6 Table, CASP8 SNPD302H and age at diagnosis. S7 Table, CASP8 SNPD302H association with Stage 4 and MYCN status. (DOCX) [file pone.0114696.s001.docx]

**Table S1:** RT-qPCR primers

| **Gene** | **Forward primer** | **Reverse primer** |
| --- | --- | --- |
| Caspase-8 | CTCCCCAAACTTGCTTTATG | AAGACCCCAGAGCATTGTTA |
| Alu-Sq | CATGGTGAAACCCCGTCTCTA | GCCTCAGCCTCCCGAGTAG |
| HMBS | GGCAATGCGGCTGCAA | GGGTACCCACGCGAATCAC |
| HPRT1 | TGACACTGGCAAAACAATGCA | GGTCCTTTTCACCAGCAAGCT |
| UBC | ATTTGGGTCGCGGTTCTTG | TGCCTTGACATTCTCGATGGT |
| SDHA | TGGGAACAAGAGGGCATCTG | CCACCACTGCATCAAATTCATG |

**Table S2**: Comparison of genotyping data on amplified DNA (A) and non-amplified DNA (NA) for 18 SNPs in 31 NB cell lines

|  | CD44-(rs187115) | | CDKN1B-(rs34330) | | YWHAQ-(rs6734469) | | CASP8- (rs1045485) | | ATM-(rs1800054) | | CCNG1-(rs2069347) | | CSE1L-(rs2426127) | | PPP2R2B-(rs319227) | | PPP2R2B-(rs319217) | | CDKN1A-  rs1801270 | | MDM4- (rs4245739) | | PIAS1-(rs1027154) | | TP53.  (rs1042522) | | TP73-  (rs1801173) | | TP73- (rs2273953) | | MDM2-(rs2279744) | | MDM2-(rs769412) | | KDR- (rs2168945) | |
| --- | --- | --- | --- | --- | --- | --- | --- | --- | --- | --- | --- | --- | --- | --- | --- | --- | --- | --- | --- | --- | --- | --- | --- | --- | --- | --- | --- | --- | --- | --- | --- | --- | --- | --- | --- | --- |
| Cell line ID | A | NA | A | NA | A | NA | A | NA | A | NA | A | NA | A | NA | A | NA | A | NA | A | NA | A | NA | A | NA | A | NA | A | NA | A | NA | A | NA | A | NA | A | NA |
| NBL-S | C/T | C/T | C/C | C/C | A/G | A/G | G/G | G/G | C/C | C/C | C/C | C/C | C/C | C/C | C/C | C/C | C/C | C/C | C/C | C/C | A/A | A/A | G/G | G/G | C/C | C/C | C/C | C/C | G/G | G/G | T/T | T/T | A/A | A/A | C/C | C/C |
| STA-NB-12 | T/T | T/T | C/C | C/C | G/G | G/G | G/G | G/G | C/C | C/C | C/C | C/C | C/C | C/C | A/C | A/C | T/C | T/C | C/C | C/C | A/A | A/A | A/G | A/G | C/C | C/C | C/C | C/C | G/G | G/G | G/G | G/G | A/A | A/A | A/A | A/A |
| NB-1 | C/T | C/T | T/T | T/T | G/G | G/G | G/G | G/G | C/C | C/C | T/T | T/T | T/T | T/T | C/C | C/C | C/C | C/C | C/C | C/C | A/A | A/A | G/G | G/G | C/G | C/G | C/C | C/C | G/G | G/G | G/T | G/T | A/G | A/G | A/A | A/A |
| SK-N-FI | T/T | T/T | C/T | C/T | A/A | A/A | G/G | G/G | C/C | C/C | C/C | C/C | C/C | C/C | A/C | A/C | T/C | T/C | C/C | C/C | A/C | A/C | G/G | G/G | C/C | C/C | C/C | C/C | G/G | G/G | G/G | G/G | A/A | A/A | A/C | A/C |
| CLB-GA | C/C | C/C | C/T | C/T | A/G | A/G | G/G | G/G | C/C | C/C | C/C | C/C | C/C | C/C | A/C | A/C | T/C | T/C | A/A | A/A | A/C | A/C | G/G | G/G | C/C | C/C | C/C | C/C | G/G | G/G | G/T | G/T | A/A | A/A | C/C | C/C |
| SK-N-BE(2c) | T/T | T/T | C/C | C/C | G/G | G/G | G/G | G/G | C/C | C/C | C/T | C/T | C/C | C/C | A/C | A/C | T/C | T/C | C/C | C/C | A/C | A/C | G/G | G/G | C/G | C/G | C/C | C/C | G/G | G/G | G/T | G/T | A/A | A/A | A/C | A/C |
| NLF | C/C | C/C | C/C | C/C | A/G | A/G | G/G | G/G | C/C | C/C | T/T | T/T | C/T | C/T | A/A | A/A | T/T | T/T | C/C | C/C | A/A | A/A | A/G | A/G | G/G | G/G | C/C | C/C | G/G | G/G | T/T | T/T | A/A | A/A | A/C | A/C |
| IMR-32 (GI-LI-N) | C/T | C/T | C/C | C/C | A/A | A/A | G/G | G/G | C/C | C/C | C/T | C/T | C/T | C/T | A/C | A/C | T/C | T/C | C/C | C/C | A/A | A/A | A/G | A/G | C/C | C/C | C/C | C/C | G/G | G/G | G/T | G/T | A/A | A/A | C/C | C/C |
| SK-N-AS | C/C | C/C | C/C | C/C | A/G | A/G | G/G | G/G | C/C | C/C | C/C | C/C | C/C | C/C | A/A | A/A | T/T | T/T | C/C | C/C | A/A | A/A | A/G | A/G | C/C | C/C | C/C | C/C | G/G | G/G | T/T | T/T | A/A | A/A | UND | UND |
| CHP 134 | T/T | T/T | C/T | C/T | A/G | A/G | G/G | G/G | C/C | C/C | C/T | C/T | C/T | C/T | A/A | A/A | T/T | T/T | A/C | A/C | A/C | A/C | A/G | A/G | C/C | C/C | T/T | T/T | A/A | A/A | G/G | G/G | A/A | A/A | A/C | A/C |
| CHP 901 | C/T | C/T | C/T | C/T | A/G | A/G | G/G | G/G | C/C | C/C | T/T | T/T | C/C | C/C | C/C | C/C | C/C | C/C | C/C | C/C | A/C | A/C | G/G | G/G | C/C | C/C | T/T | T/T | A/A | A/A | G/T | G/T | A/A | A/A | A/C | A/C |
| N206 | C/T | C/T | T/T | T/T | A/A | A/A | G/G | G/G | C/C | C/C | T/T | T/T | C/C | C/C | C/C | C/C | C/C | C/C | C/C | C/C | A/C | A/C | G/G | G/G | C/C | C/C | T/T | T/T | A/A | A/A | G/T | G/T | A/A | A/A | A/C | A/C |
| SJNB-8 | T/T | T/T | C/C | C/C | G/G | G/G | G/G | G/G | C/C | C/C | T/T | T/T | T/T | T/T | A/C | A/C | T/C | T/C | A/A | A/A | A/A | A/A | G/G | G/G | G/G | G/G | C/C | C/C | G/G | G/G | G/G | G/G | A/A | A/A | A/A | A/A |
| SJNB-6 | T/T | T/T | C/C | C/C | A/G | A/G | G/G | G/G | C/C | C/C | T/T | T/T | C/C | C/C | A/C | A/C | T/C | T/C | A/C | A/C | A/A | A/A | G/G | G/G | G/G | G/G | C/C | C/C | G/G | G/G | T/T | T/T | A/A | A/A | A/A | A/A |
| GI-ME-N | UND | UND | C/C | C/C | A/A | A/A | G/G | G/G | C/C | C/C | T/T | T/T | C/C | C/C | C/C | C/C | C/C | C/C | A/C | A/C | A/A | A/A | G/G | G/G | G/G | G/G | C/C | C/C | G/G | G/G | T/T | T/T | A/G | A/G | A/C | A/C |
| SH-SY5Y | C/T | C/T | C/T | C/T | G/G | G/G | G/G | G/G | C/C | C/C | C/T | C/T | C/C | C/C | A/C | A/C | T/C | T/C | C/C | C/C | A/C | A/C | A/G | A/G | G/G | G/G | C/C | C/C | G/G | G/G | T/T | T/T | A/A | A/A | A/C | A/C |
| SH-EP | C/T | C/T | C/T | C/T | G/G | G/G | C/G | C/G | C/C | C/C | C/T | C/T | C/T | C/T | A/A | A/A | T/T | T/T | C/C | C/C | C/C | C/C | A/G | A/G | C/C | C/C | C/C | C/C | G/G | G/G | T/T | T/T | A/A | A/A | C/C | C/C |
| NGP | C/C | C/C | T/T | T/T | A/G | A/G | C/G | C/G | C/C | C/C | T/T | T/T | C/T | C/T | C/C | C/C | C/C | C/C | C/C | C/C | UND | UND | G/G | G/G | G/G | G/G | C/C | C/C | G/G | G/G | T/T | T/T | A/A | A/A | A/A | A/A |
| SK-N-BE | T/T | T/T | C/C | C/C | G/G | G/G | G/G | G/G | C/C | C/C | C/C | C/C | T/T | T/T | A/C | A/C | T/C | T/C | C/C | C/C | A/C | A/C | A/G | A/G | C/C | C/C | C/T | C/T | UND | UND | G/T | G/T | A/A | A/A | C/C | C/C |
| CHP 902R | C/T | C/T | C/C | C/C | A/G | A/G | G/G | G/G | C/C | C/C | C/C | C/C | T/T | T/T | A/C | A/C | T/C | T/C | C/C | C/C | A/C | A/C | A/G | A/G | C/C | C/C | C/T | C/T | G/A | G/A | G/T | G/T | A/A | A/A | C/C | C/C |
| SK-N-SH | C/T | C/T | C/T | C/T | G/G | G/G | G/G | G/G | C/C | C/C | UND | UND | T/T | T/T | A/A | A/A | T/T | T/T | C/C | C/C | A/C | A/C | G/G | G/G | C/G | C/G | C/C | C/C | G/G | G/G | G/T | G/T | A/A | A/A | UND | UND |
| SJNB-10 | C/T | C/T | C/C | C/C | A/G | A/G | G/G | G/G | C/C | C/C | C/T | C/T | C/C | C/C | A/C | A/C | T/C | T/C | C/C | C/C | C/C | C/C | G/G | G/G | C/C | C/C | C/C | C/C | G/G | G/G | T/T | T/T | A/A | A/A | A/C | A/C |
| NMB | C/T | C/T | T/T | T/T | A/A | A/A | G/G | G/G | C/C | C/C | C/T | C/T | C/T | C/T | A/C | A/C | T/C | T/C | C/C | C/C | A/A | A/A | A/G | A/G | G/G | G/G | C/C | C/C | G/G | G/G | G/G | G/G | A/A | A/A | A/C | A/C |
| TR-14 | T/T | T/T | C/T | C/T | A/G | A/G | G/G | G/G | C/C | C/C | C/T | C/T | C/T | C/T | A/A | A/A | T/T | T/T | UND | UND | A/C | A/C | G/G | G/G | C/C | C/C | C/C | C/C | G/G | G/G | T/T | T/T | A/G | A/G | A/C | A/C |
| SMS-KCNR | C/T | C/T | C/C | C/C | A/G | A/G | G/G | G/G | C/C | C/C | C/C | C/C | T/T | T/T | A/C | A/C | T/C | T/C | C/C | C/C | A/C | A/C | A/G | A/G | C/C | C/C | C/T | C/T | G/A | G/A | G/T | G/T | A/A | A/A | C/C | C/C |
| Gi-CIN-1 | T/T | T/T | C/C | C/C | A/A | A/A | G/G | G/G | C/C | C/C | T/T | T/T | C/C | C/C | A/C | A/C | T/C | T/C | C/C | C/C | A/A | A/A | G/G | G/G | C/C | C/C | C/C | C/C | G/G | G/G | G/G | G/G | A/A | A/A | A/A | A/A |
| STA-NB-10 | T/T | T/T | C/C | C/C | A/A | A/A | G/G | G/G | C/C | C/C | T/T | T/T | C/C | C/C | A/C | A/C | T/C | T/C | C/C | C/C | A/A | A/A | G/G | G/G | C/C | C/C | C/C | C/C | G/G | G/G | G/G | G/G | A/A | A/A | A/A | A/A |
| SJNB-12 | C/C | C/C | C/T | C/T | G/G | G/G | C/G | C/G | C/C | C/C | C/C | C/C | C/T | C/T | C/C | C/C | C/C | C/C | A/C | A/C | A/C | A/C | G/G | G/G | C/C | C/C | C/C | C/C | G/G | G/G | UND | UND | A/A | A/A | A/C | A/C |
| SMS-KAN | C/T | C/T | C/T | C/T | G/G | G/G | G/G | G/G | C/C | C/C | C/T | C/T | C/T | C/T | A/C | A/C | T/C | T/C | A/C | A/C | A/A | A/A | A/G | A/G | C/C | C/C | C/C | C/C | G/G | G/G | T/T | T/T | A/A | A/A | A/A | A/A |
| LA-N-5 | C/C | C/C | C/C | C/C | A/G | A/G | C/G | C/G | C/C | C/C | C/C | C/C | C/C | C/C | A/A | A/A | T/T | T/T | C/C | C/C | A/C | A/C | G/G | G/G | C/C | C/C | T/T | T/T | A/A | A/A | T/T | T/T | A/A | A/A | A/A | A/A |
| UKF-NB-3 | C/T | C/T | UND | UND | A/A | A/A | C/G | C/G | C/C | C/C | C/C | C/C | C/C | C/C | A/A | A/A | T/T | T/T | C/C | C/C | A/C | A/C | G/G | G/G | C/C | C/C | T/T | T/T | A/A | A/A | T/T | T/T | A/A | A/A | A/A | A/A |
| STA-NB-8 | T/T | T/T | C/T | C/T | G/G | G/G | C/C | C/C | C/C | C/C | C/T | C/T | T/T | T/T | A/A | A/A | T/T | T/T | C/C | C/C | A/C | A/C | G/G | G/G | C/G | C/G | C/C | C/C | G/G | G/G | T/T | T/T | A/A | A/A | C/C | C/C |
| STA-NB-3 | T/T | T/T | C/C | C/C | G/G | G/G | G/G | G/G | C/C | C/C | C/T | C/T | C/C | C/C | C/C | C/C | C/C | C/C | C/C | C/C | A/A | A/A | A/G | A/G | C/C | C/C | T/T | T/T | A/A | A/A | G/T | G/T | A/A | A/A | UND | UND |
| SKMYC2 | C/T | C/T | C/T | C/T | G/G | G/G | G/G | G/G | C/C | C/C | T/T | T/T | C/T | C/T | C/C | C/C | C/C | C/C | C/C | C/C | A/A | A/A | G/G | G/G | C/C | C/C | C/C | C/C | G/G | G/G | G/T | G/T | A/A | A/A | A/A | A/A |
| STA-NB-9 | C/T | C/T | C/C | C/C | A/A | A/A | G/G | G/G | C/G | C/G | C/T | C/T | C/C | C/C | A/C | A/C | T/C | T/C | C/C | C/C | A/C | A/C | G/G | G/G | C/G | C/G | C/C | C/C | G/G | G/G | T/T | T/T | A/A | A/A | A/A | A/A |
| LA-N-2 | C/T | C/T | C/C | C/C | A/A | A/A | G/G | G/G | C/C | C/C | C/C | C/C | C/C | C/C | A/C | A/C | T/C | T/C | C/C | C/C | A/A | A/A | G/G | G/G | C/C | C/C | C/C | C/C | G/G | G/G | T/T | T/T | A/A | A/A | A/A | A/A |
| NB-13 | C/T | C/T | C/T | C/T | A/A | A/A | G/G | G/G | C/C | C/C | T/T | T/T | C/C | C/C | A/C | A/C | T/C | T/C | UND | UND | A/A | A/A | G/G | G/G | C/C | C/C | C/C | C/C | G/G | G/G | T/T | T/T | A/A | A/A | UND | UND |
| ACN | C/C | C/C | UND | UND | A/A | A/A | C/G | C/G | C/C | C/C | C/C | C/C | C/C | C/C | A/A | A/A | T/T | T/T | C/C | C/C | A/C | A/C | G/G | G/G | C/G | C/G | C/C | C/C | UND | UND | G/T | G/T | A/A | A/A | A/A | A/A |
| Kelly | C/T | C/T | T/T | T/T | A/A | A/A | G/G | G/G | C/C | C/C | C/T | C/T | C/C | C/C | A/A | A/A | T/T | T/T | C/C | C/C | A/A | A/A | G/G | G/G | C/C | C/C | C/C | C/C | G/G | G/G | G/G | G/G | A/A | A/A | A/C | A/C |
| NB-5 | C/T | C/T | C/T | C/T | A/A | A/A | G/G | G/G | C/C | C/C | C/C | C/C | C/C | C/C | C/C | C/C | C/C | C/C | A/A | A/A | A/C | A/C | G/G | G/G | C/G | C/G | C/C | C/C | G/G | G/G | T/T | T/T | A/A | A/A | A/C | A/C |
| UHG-NP | C/T | C/T | T/T | T/T | UND | UND | C/G | C/G | C/C | C/C | T/T | T/T | C/C | C/C | A/C | A/C | T/C | T/C | C/C | C/C | C/C | C/C | G/G | G/G | C/C | C/C | C/C | C/C | G/G | G/G | T/T | T/T | A/A | A/A | A/A | A/A |
| LAN-6 | T/T | T/T | T/T | T/T | A/G | A/G | G/G | G/G | C/C | C/C | C/T | C/T | C/C | C/C | A/C | A/C | T/C | T/C | C/C | C/C | A/C | A/C | G/G | G/G | C/G | C/G | T/T | T/T | A/A | A/A | T/T | T/T | A/A | A/A | C/C | C/C |
| CHLA-90 | C/T | C/T | C/C | C/C | A/G | A/G | G/G | G/G | C/C | C/C | C/C | C/C | T/T | T/T | A/C | A/C | T/C | T/C | C/C | C/C | A/C | A/C | A/G | A/G | C/C | C/C | C/T | C/T | G/A | G/A | G/T | G/T | A/A | A/A | C/C | C/C |
| LAN-6 | T/T | T/T | T/T | T/T | A/G | A/G | G/G | G/G | C/C | C/C | T/T | T/T | C/T | C/T | A/A | A/A | T/T | T/T | C/C | C/C | A/A | A/A | A/G | A/G | G/G | G/G | C/C | C/C | G/G | G/G | T/T | T/T | A/A | A/A | UND | UND |
| LA-N-1 | T/T | T/T | C/T | C/T | A/G | A/G | G/G | G/G | C/C | C/C | T/T | T/T | C/T | C/T | A/A | A/A | T/T | T/T | C/C | C/C | A/A | A/A | G/G | G/G | C/C | C/C | C/C | C/C | G/G | G/G | T/T | T/T | A/A | A/A | A/A | A/A |
| SMS-KCN | C/T | C/T | C/C | C/C | A/G | A/G | G/G | G/G | C/C | C/C | C/C | C/C | C/T | C/T | C/C | C/C | C/C | C/C | C/C | C/C | UND | UND | G/G | G/G | C/C | C/C | C/C | C/C | G/G | G/G | G/G | G/G | A/A | A/A | A/C | A/C |
| UKF-NB-2 | C/T | C/T | C/C | UND | A/G | A/G | G/G | G/G | C/C | C/C | C/T | C/T | UND | C/T | A/A | A/A | T/T | T/T | C/C | C/C | A/C | A/C | G/G | G/G | C/C | C/C | C/C | C/C | G/G | G/G | T/T | T/T | A/G | A/G | A/A | A/A |

**Table S3**: Stratified survival analysis of all the SNPs

| **Gene symbol** | **Reference SNP ID** | **Stratification** | **Analysis** | ***p*-values** |
| --- | --- | --- | --- | --- |
| ATM | rs1800054 | All | EFS | 0,07 |
| ATM | rs1800054 | MNA | EFS | 0,393 |
| ATM | rs1800054 | MNN | EFS | 0,0317 |
| ATM | rs1800054 | Stage 4 | EFS | 0,3584 |
| ATM | rs1800054 | Stages (1,2,3,4s) | EFS | 0,0255 |
| ATM | rs1800054 | All | OS | 0,5256 |
| ATM | rs1800054 | MNA | OS | 0,9232 |
| ATM | rs1800054 | MNN | OS | 0,226 |
| ATM | rs1800054 | Stage 4 | OS | 0,9612 |
| ATM | rs1800054 | Stages (1,2,3,4s) | OS | 0,0889 |
| CCNG1 | rs2069347 | All | EFS | 0,9385 |
| CCNG1 | rs2069347 | MNA | EFS | 0,4201 |
| CCNG1 | rs2069347 | MNN | EFS | 0,8379 |
| CCNG1 | rs2069347 | Stage 4 | EFS | 0,5452 |
| CCNG1 | rs2069347 | Stages (1,2,3,4s) | EFS | 0,0364 |
| CCNG1 | rs2069347 | All | OS | 0,6189 |
| CCNG1 | rs2069347 | MNA | OS | 0,3129 |
| CCNG1 | rs2069347 | MNN | OS | 0,5626 |
| CCNG1 | rs2069347 | Stage 4 | OS | 0,5248 |
| CCNG1 | rs2069347 | Stages (1,2,3,4s) | OS | 0,0441 |
| CD44 | rs187115 | All | EFS | 0,734 |
| CD44 | rs187115 | MNA | EFS | 0,3229 |
| CD44 | rs187115 | MNN | EFS | 0,9458 |
| CD44 | rs187115 | Stage 4 | EFS | 0,5833 |
| CD44 | rs187115 | Stages (1,2,3,4s) | EFS | 0,7536 |
| CD44 | rs187115 | All | OS | 0,195 |
| CD44 | rs187115 | MNA | OS | 0,5173 |
| CD44 | rs187115 | MNN | OS | 0,1054 |
| CD44 | rs187115 | Stage 4 | OS | 0,1517 |
| CD44 | rs187115 | Stages (1,2,3,4s) | OS | 0,3043 |
| CDKN1A | rs1801270 | All | EFS | 0,2402 |
| CDKN1A | rs1801270 | MNA | EFS | 0,7292 |
| CDKN1A | rs1801270 | MNN | EFS | 0,1168 |
| CDKN1A | rs1801270 | Stage 4 | EFS | 0,4957 |
| CDKN1A | rs1801270 | Stages (1,2,3,4s) | EFS | 0,713 |
| CDKN1A | rs1801270 | All | OS | 0,6538 |
| CDKN1A | rs1801270 | MNA | OS | 0,6873 |
| CDKN1A | rs1801270 | MNN | OS | 0,5547 |
| CDKN1A | rs1801270 | Stage 4 | OS | 0,7144 |
| CDKN1A | rs1801270 | Stages (1,2,3,4s) | OS | 0,3124 |
| CDKN1B | rs34330 | All | EFS | 0,9628 |
| CDKN1B | rs34330 | MNA | EFS | 0,4233 |
| CDKN1B | rs34330 | MNN | EFS | 0,7346 |
| CDKN1B | rs34330 | Stage 4 | EFS | 0,9379 |
| CDKN1B | rs34330 | Stages (1,2,3,4s) | EFS | 0,3025 |
| CDKN1B | rs34330 | All | OS | 0,3784 |
| CDKN1B | rs34330 | MNA | OS | 0,5275 |
|  |  |  |  |  |
|  |  |  |  |  |
| CDKN1B | rs34330 | MNN | OS | 0,4348 |
| CDKN1B | rs34330 | Stage 4 | OS | 0,8551 |
| CDKN1B | rs34330 | Stages (1,2,3,4s) | OS | 0,6166 |
| CSE1L | rs2426127 | All | EFS | 0,3616 |
| CSE1L | rs2426127 | MNA | EFS | 0,0888 |
| CSE1L | rs2426127 | MNN | EFS | 0,5586 |
| CSE1L | rs2426127 | Stage 4 | EFS | 0,7714 |
| CSE1L | rs2426127 | Stages (1,2,3,4s) | EFS | 0,234 |
| CSE1L | rs2426127 | All | OS | 0,5951 |
| CSE1L | rs2426127 | MNA | OS | 0,329 |
| CSE1L | rs2426127 | MNN | OS | 0,4115 |
| CSE1L | rs2426127 | Stage 4 | OS | 0,9602 |
| CSE1L | rs2426127 | Stages (1,2,3,4s) | OS | 0,2916 |
| KDR | rs2168945 | All | EFS | 0,218 |
| KDR | rs2168945 | MNA | EFS | 0,5536 |
| KDR | rs2168945 | MNN | EFS | 0,0062 |
| KDR | rs2168945 | Stage 4 | EFS | 0,8568 |
| KDR | rs2168945 | Stages (1,2,3,4s) | EFS | 0,2839 |
| KDR | rs2168945 | All | OS | 0,4777 |
| KDR | rs2168945 | MNA | OS | 0,8128 |
| KDR | rs2168945 | MNN | OS | 0,1114 |
| KDR | rs2168945 | Stage 4 | OS | 0,8187 |
| KDR | rs2168945 | Stages (1,2,3,4s) | OS | 0,8514 |
| MDM2 | rs117039649 | All | EFS | 0,9695 |
| MDM2 | rs117039649 | MNA | EFS | 0,4118 |
| MDM2 | rs117039649 | MNN | EFS | 0,5624 |
| MDM2 | rs117039649 | Stage 4 | EFS | 0,6618 |
| MDM2 | rs117039649 | Stages (1,2,3,4s) | EFS | 0,8403 |
| MDM2 | rs117039649 | All | OS | 0,9847 |
| MDM2 | rs117039649 | MNA | OS | 0,639 |
| MDM2 | rs117039649 | MNN | OS | 0,6433 |
| MDM2 | rs117039649 | Stage 4 | OS | 0,4895 |
| MDM2 | rs117039649 | Stages (1,2,3,4s) | OS | 0,3089 |
| MDM2 | rs1196333 | All | EFS | 0,0368 |
| MDM2 | rs1196333 | MNA | EFS | 0,2389 |
| MDM2 | rs1196333 | MNN | EFS | 0,001 |
| MDM2 | rs1196333 | Stage 4 | EFS | 0,3851 |
| MDM2 | rs1196333 | Stages (1,2,3,4s) | EFS | 0,0007 |
| MDM2 | rs1196333 | All | OS | 0,3608 |
| MDM2 | rs1196333 | MNA | OS | 0,1631 |
| MDM2 | rs1196333 | MNN | OS | 0,0348 |
| MDM2 | rs1196333 | Stage 4 | OS | 0,2122 |
| MDM2 | rs1196333 | Stages (1,2,3,4s) | OS | 0,0865 |
| MDM2 | rs769412 | All | EFS | 0,5011 |
| MDM2 | rs769412 | MNA | EFS | 0,0968 |
| MDM2 | rs769412 | MNN | EFS | 0,1912 |
| MDM2 | rs769412 | Stage 4 | EFS | 0,8293 |
| MDM2 | rs769412 | Stages (1,2,3,4s) | EFS | 0,2071 |
| MDM2 | rs769412 | All | OS | 0,8096 |
| MDM2 | rs769412 | MNA | OS | 0,0248 |
| MDM2 | rs769412 | MNN | OS | 0,5211 |
| MDM2 | rs769412 | Stage 4 | OS | 0,5283 |
| MDM2 | rs769412 | Stages (1,2,3,4s) | OS | 0,6858 |
| MDM2 | rs2279744 | All | EFS | 0,483 |
| MDM2 | rs2279744 | MNA | EFS | 0,4683 |
| MDM2 | rs2279744 | MNN | EFS | 0,8639 |
| MDM2 | rs2279744 | Stage 4 | EFS | 0,1455 |
| MDM2 | rs2279744 | Stages (1,2,3,4s) | EFS | 0,4184 |
| MDM2 | rs2279744 | All | OS | 0,6206 |
| MDM2 | rs2279744 | MNA | OS | 0,6345 |
| MDM2 | rs2279744 | MNN | OS | 0,6814 |
| MDM2 | rs2279744 | Stage 4 | OS | 0,0717 |
| MDM2 | rs2279744 | Stages (1,2,3,4s) | OS | 0,955 |
| MDM4 | rs4245739 | All | EFS | 0,1416 |
| MDM4 | rs4245739 | MNA | EFS | 0,7989 |
| MDM4 | rs4245739 | MNN | EFS | 0,3338 |
| MDM4 | rs4245739 | Stage 4 | EFS | 0,5483 |
| MDM4 | rs4245739 | Stages (1,2,3,4s) | EFS | 0,056 |
| MDM4 | rs4245739 | All | OS | 0,2853 |
| MDM4 | rs4245739 | MNA | OS | 0,6707 |
| MDM4 | rs4245739 | MNN | OS | 0,6989 |
| MDM4 | rs4245739 | Stage 4 | OS | 0,7991 |
| MDM4 | rs4245739 | Stages (1,2,3,4s) | OS | 0,2365 |
| p53 | rs78378222 | All | EFS | 0,5534 |
| p53 | rs78378222 | MNA | EFS | 0,1824 |
| p53 | rs78378222 | MNN | EFS | 0,1182 |
| p53 | rs78378222 | Stage 4 | EFS | 0,6113 |
| p53 | rs78378222 | Stages (1,2,3,4s) | EFS | 0,5368 |
| p53 | rs78378222 | All | OS | 0,9917 |
| p53 | rs78378222 | MNA | OS | 0,394 |
| p53 | rs78378222 | MNN | OS | 0,4494 |
| p53 | rs78378222 | Stage 4 | OS | 0,8734 |
| p53 | rs78378222 | Stages (1,2,3,4s) | OS | 0,7256 |
| p73 | rs1801173 | All | EFS | 0,3289 |
| p73 | rs1801173 | MNA | EFS | 0,2715 |
| p73 | rs1801173 | MNN | EFS | 0,837 |
| p73 | rs1801173 | Stage 4 | EFS | 0,4304 |
| p73 | rs1801173 | Stages (1,2,3,4s) | EFS | 0,639 |
| p73 | rs1801173 | All | OS | 0,478 |
| p73 | rs1801173 | MNA | OS | 0,296 |
| p73 | rs1801173 | MNN | OS | 0,8102 |
| p73 | rs1801173 | Stage 4 | OS | 0,7661 |
| p73 | rs1801173 | Stages (1,2,3,4s) | OS | 0,493 |
| p73 | rs2273953 | All | EFS | 0,271 |
| p73 | rs2273953 | MNA | EFS | 0,2817 |
| p73 | rs2273953 | MNN | EFS | 0,5486 |
| p73 | rs2273953 | Stage 4 | EFS | 0,5216 |
| p73 | rs2273953 | Stages (1,2,3,4s) | EFS | 0,6757 |
| p73 | rs2273953 | All | OS | 0,6411 |
| p73 | rs2273953 | MNA | OS | 0,2377 |
| p73 | rs2273953 | MNN | OS | 0,5964 |
| p73 | rs2273953 | Stage 4 | OS | 0,8439 |
| p73 | rs2273953 | Stages (1,2,3,4s) | OS | 0,3333 |
| PIAS | rs1027154 | All | EFS | 0,9631 |
| PIAS | rs1027154 | MNA | EFS | 0,2966 |
| PIAS | rs1027154 | MNN | EFS | 0,9937 |
| PIAS | rs1027154 | Stage 4 | EFS | 0,7314 |
| PIAS | rs1027154 | Stages (1,2,3,4s) | EFS | 0,4284 |
| PIAS | rs1027154 | All | OS | 0,657 |
| PIAS | rs1027154 | MNA | OS | 0,4922 |
| PIAS | rs1027154 | MNN | OS | 0,7318 |
| PIAS | rs1027154 | Stage 4 | OS | 0,8697 |
| PIAS | rs1027154 | Stages (1,2,3,4s) | OS | 0,209 |
| PPP2R2B | rs319217 | All | EFS | 0,5665 |
| PPP2R2B | rs319217 | MNA | EFS | 0,9523 |
| PPP2R2B | rs319217 | MNN | EFS | 0,5765 |
| PPP2R2B | rs319217 | Stage 4 | EFS | 0,395 |
| PPP2R2B | rs319217 | Stages (1,2,3,4s) | EFS | 0,5461 |
| PPP2R2B | rs319217 | All | OS | 0,5457 |
| PPP2R2B | rs319217 | MNA | OS | 0,4607 |
| PPP2R2B | rs319217 | MNN | OS | 0,7725 |
| PPP2R2B | rs319217 | Stage 4 | OS | 0,336 |
| PPP2R2B | rs319217 | Stages (1,2,3,4s) | OS | 0,2005 |
| PPP2R2B | rs319227 | All | EFS | 0,517 |
| PPP2R2B | rs319227 | MNA | EFS | 0,3106 |
| PPP2R2B | rs319227 | MNN | EFS | 0,5165 |
| PPP2R2B | rs319227 | Stage 4 | EFS | 0,6581 |
| PPP2R2B | rs319227 | Stages (1,2,3,4s) | EFS | 0,4968 |
| PPP2R2B | rs319227 | All | OS | 0,54 |
| PPP2R2B | rs319227 | MNA | OS | 0,9028 |
| PPP2R2B | rs319227 | MNN | OS | 0,7628 |
| PPP2R2B | rs319227 | Stage 4 | OS | 0,4084 |
| PPP2R2B | rs319227 | Stages (1,2,3,4s) | OS | 0,4798 |
| YWHAQ | rs6734469 | All | EFS | 0,3986 |
| YWHAQ | rs6734469 | MNA | EFS | 0,2076 |
| YWHAQ | rs6734469 | MNN | EFS | 0,4171 |
| YWHAQ | rs6734469 | Stage 4 | EFS | 0,1148 |
| YWHAQ | rs6734469 | Stages (1,2,3,4s) | EFS | 0,4896 |
| YWHAQ | rs6734469 | All | OS | 0,4163 |
| YWHAQ | rs6734469 | MNA | OS | 0,9281 |
| YWHAQ | rs6734469 | MNN | OS | 0,6211 |
| YWHAQ | rs6734469 | Stage 4 | OS | 0,681 |
| YWHAQ | rs6734469 | Stages (1,2,3,4s) | OS | 0,2527 |

**Table S4**: multivariate cox proportional hazards analysis (overall survival data)

The variables in the equation include *CASP8* SNP D302H, age at diagnosis, *MYCN* status, and stage of the disease. The analysis was performed according to the “Enter” method in SPSS v. 20. B is the cox regression-model coefficient. Exp(B) is the hazard ratio.

**Table S5**: multivariate proportional hazards analysis (event-free survival data)

| Event-free survival | B | *P*-value | exp(B) | 95.0% CI for exp(B) | |
| --- | --- | --- | --- | --- | --- |
|  |  |  |  | Lower | Upper |
| *CASP8* SNP D302H (CC+CG vs. GG) | 0.495 | 0.008 | 1.640 | 1.137 | 2.366 |
| Age at diagnosis (>12 months vs. <12 months) | 0.772 | 0.0009 | 2.164 | 1.369 | 3.421 |
| *MYCN* (amplified vs. non amplified) | 0.942 | 4.4E-08 | 2.564 | 1.830 | 3.592 |
| Stage [4 vs. (1, 2, 3, 4S)] | 1.592 | 1.8E-16 | 4.913 | 3.363 | 7.179 |

| Overall survival | B | *P*-value | exp(B) | 95.0% CI for exp(B) |  |
| --- | --- | --- | --- | --- | --- |
|  |  |  |  | Lower | Upper |
| *CASP8* SNP D302H (CC+CG vs. GG) | 0.684 | 0.001 | 1.982 | 1.313 | 2.991 |
| Age at diagnosis (>12 months vs. <12 months) | 1.355 | 0.0001 | 3.876 | 1.954 | 7.686 |
| *MYCN* (amplified vs. non amplified) | 1.110 | 1.1E-08 | 3.036 | 2.074 | 4.444 |
| Stage [4 vs. (1, 2, 3, 4S)] | 2.065 | 2E-15 | 7.885 | 4.737 | 13.127 |

The variables in the equation include *CASP8* SNP D302H, age at diagnosis, *MYCN* status, and stage of the disease. The analysis was performed according to the “Enter” method in SPSS v. 20. B is the cox regression-model coefficient. Exp(B) is the hazard ratio

|  |  | GG | |  | | CC/CG | | | |  | |  | |  | |  |  |
| --- | --- | --- | --- | --- | --- | --- | --- | --- | --- | --- | --- | --- | --- | --- | --- | --- | --- |
| *CASP* D302H |  | N | Mean age | |  | | N | Mean age |  | | *p*-values | |  | | *q*-values | | |
| All stages |  | 409 | 26 | |  | | 91 | 26.5 |  | | 0.4 | |  | | 0.96 | | |
| Stage 4 |  | 145 | 23.9 | |  | | 38 | 22.1 |  | | 0.51 | |  | | 0.96 | | |
| Stages (1,2,3,4s) |  | 264 | 27.4 | |  | | 53 | 29.7 |  | | 0.055 | |  | | 0.83 | | |
| MNA |  | 73 | 30.3 | |  | | 21 | 30.5 |  | | 0.605 | |  | | 0.962 | | |
| MNN |  | 336 | 25.6 | |  | | 70 | 3.08 |  | | 0.282 | |  | | 0.956 | | |

**Table S6**: *CASP8* SNPD302H and age at diagnosis

*q*-value is the adjusted *p*-value after Benjamini-Hochberg multiple testing correction

Raw *P*-value is calculated by Mann-Whitney *U* test. Mean age is the mean age at diagnosis in months.

**Table S7**:  *CASP8* SNPD302H association with Stage 4 and *MYCN* status

|  | *CASP8* SNPD302H | |  |  |  |  |  | |
| --- | --- | --- | --- | --- | --- | --- | --- | --- |
|  | GG | CC/CG | | Total |  | *p*-values | | *q*-values |
| Stage 4 | 145 | 38 | | 183 |  | 0.2 | | 0.956 |
| Stages (1,2,3,4s) | 264 | 53 | | 317 |  |  |  |  |
| MNA | 73 | 21 | | 94 |  | 0.26 | | 0.956 |
| MNN | 336 | 70 | | 406 |  |  |  |  |
| Total | 409 | 91 | | 500 |  |  | |  |

Raw *P*-value is calculated by Pearson Chi-Square test

*q*-value is the adjusted *p*-value after Benjamini-Hochberg multiple testing correction
